# Miniplegia versus blood cardioplegia in elective aortic valve replacement: a prospective randomised, non - inferiority controlled trial

**DOI:** 10.1186/1749-8090-10-S1-A60

**Published:** 2015-12-16

**Authors:** Eduardo Bernabeu, Antonio García-Valentín, Juan Meseguer, Aquilino Hurlé, Patricio Llamas

**Affiliations:** 1 Dept. Cardiac Surgery, Hospital General Universitario de Alicante, Alicante, E-03010, Spain

## Background/Introduction

Antegrade intermittent 4:1 blood cardioplegia with Buckberg solution is widely used in elective aortic valve replacement. Use of miniplegia could simplify myocardial protection in this setting.

## Aims/Objectives

Our objective was to compare both strategies in terms of non-inferiority.

## Method

A prospective, randomised controlled trial was performed. Primary end-point was demonstrating non-inferiority of miniplegia versus intermittent 4:1 blood cardioplegia in elective aortic valve replacement. For sample size calculation, a maximum increase +15% in mean peak postoperative troponin T was considered non-inferior (Δ = +474.24 ng/L). Power was 0.9, and α < 0.05 was considered statistically significant. Secondary end-points were differences in troponin curve, reperfusion and postoperative rhythm, haematocrit, use of inotropic and vasopressor drug support, ICU stay, and postoperative evolution.

## Results

66 patients were enrolled and randomised. There were no significant differences in baseline and preoperative variables. Peak troponin T in miniplegia group was non-inferior to blood cardioplegia group (p = 0.036). Patients in the miniplegia group showed a higher incidence of spontaneous sinus rhythm after myocardial ischemia (18/33, 54.5% versus 8/33, 24.2%, p = 0.005) and fewer patients required defibrillation (9/33, 27.7% versus 21/33, 63.6%, p = 0.03) for ventricular reperfusion arrhythmias. Postoperatively, there were no differences in troponin T release, inotropic and vasopressor drug support, ICU stay, and postoperative mortality.

## Discussion/Conclusion

Miniplegia used as myocardial protection in elective aortic replacement is non-inferior to blood cardioplegia. Preferential return to sinus rhythm and lower incidence of reperfusion arrhythmias in the miniplegia group could reflect a better myocardial protection during cardioplegic arrest. Ease of administration and inexpensive use of miniplegia are additional benefits.

